# Screening of Fungicides and Comparison of Selective Media for Isolation of *Fusarium graminearum* from Soil and Plant Material

**DOI:** 10.3390/pathogens12020197

**Published:** 2023-01-28

**Authors:** Samina Ashiq, Matthew Back, Andrew Watson, Simon G. Edwards

**Affiliations:** Agriculture and Environment Department, Harper Adams University, Newport TF10 8NB, UK

**Keywords:** fluxapyroxad, Czapek Dox propiconazole dichloran agar, Komada, Fusarium head blight, wheat

## Abstract

The culture media recommended for the isolation and enumeration of the *Fusarium* spp. lack selectivity for *Fusarium graminearum*. Five fungicides—Amistar^®^ (250 g·L^−1^ azoxystrobin), Filan^®^ (500 g·kg^−1^ boscalid), Comet^®^ 200 (200 g·L^−1^ pyraclostrobin), Imtrex^®^ (62.5 g·L^−1^ fluxapyroxad), Poraz^®^ (450 g·L^−1^ prochloraz)—were investigated for their potential as selective inhibitors in culture media for the isolation of *F. graminearum* from soil and plant material. Based on the screening, fluxapyroxad was further tested for selective inhibition for the isolation of *F. graminearum* from soil. Additionally, selective media were compared for the isolation of *F. graminearum* from plant material. The fungicides tested did not prove to be effective inhibitors for the development of selective media. For the detection of *F. graminearum* in plant material, Czapek Dox propiconazole dichloran agar was found to be a better medium than Komada’s media, as the former resulted in colonies with darker pigmentation over a shorter incubation time and appeared to have a less inhibitory effect on *F. graminearum* growth.

## 1. Introduction

A number of selective media have been developed for the isolation and enumeration of the *Fusarium* species. The most widely used selective medium for *Fusarium* is the Nash and Snyder medium [1] which contains pentachloronitrobenzene (PCNB) as the selective agent. Other PCNB-containing media selective for *Fusarium* are the Papavizas medium [2], Komada’s medium [3] and Rose Bengal-glycerine-urea medium [4]. Due to concern about the safety of PCNB, other alternative selective inhibitors for *Fusarium* isolation have been reported such as dichloran in dichloran-chloramphenicol peptone agar [5] or a combination of iprodione and dichloran in Czapek Dox iprodione dichloran agar (CZID) [6] and potato dextrose iprodione dichloran agar [7]. Similarly, Malachite Green agar 2.5 ppm is reported to be a useful selective medium for the *Fusarium* species as an alternative to PCNB-containing media [8,9]. 

*Fusarium graminearum* is a globally important cereal pathogen that causes head blight in wheat, resulting in 50–70% yield losses [10,11]. In 2015/16, Fusarium head blight caused yield losses valued at USD 1.176 billion in the U.S [12]. Additionally, *F. graminearum* causes economic and health losses due to the production of mycotoxins, deoxynivalenol and zearalenone in cereals [13]. For the determination of the incidence of the disease and other research purposes such as understanding the disease mechanism and looking for better control strategies, it is important to isolate and identify *F. graminearum*. The isolation of *F. graminearum* on Nash and Snyder medium requires the subculture of the fungus onto potato dextrose agar (PDA) medium as the former medium often fails to maintain the morphological characteristics of *F. graminearum* [14]. Malachite Green agar 2.5 ppm supplemented with carnation leaf pieces is recommended as a semi-selective medium for *F. graminearum*, *F. proliferatum*, *F. subglutinans* and *F. verticillioides* in maize seeds [15]. A selective medium for *F. graminearum* was developed using the bacterial toxin, toxoflavin, as the selective agent [16]. This toxin is produced by the rice pathogen *Burkholderia glumae* and is inhibitory against many fungi including *Aspergillus*, *Colletotrichum* and *Penicillium*. The *Fusarium* species, particularly *F. graminearum*, was found to be highly resistant to this toxin. However, due to the high cost of toxoflavin, it is not practical for use in a selective medium.

These media are useful either for identifying *Fusarium* at the genus level or as a semi-selective media for the *Fusarium* species. However, in order to isolate *F. graminearum* from soil and plant debris, a highly selective medium is required because these types of samples have a high abundance of other fungal flora. The selective media currently used for the isolation of *Fusarium* are based on old fungicide chemistry, while newer fungicides such as strobilurins and succinate dehydrogenase inhibitors (SDHI) are less effective against *F. graminearum* [17,18]. Therefore, the purpose this research was to investigate whether these newer fungicides would improve the selectivity of *Fusarium* media. Consequently, we investigated a range of fungicides as selective inhibitors in culture media for the isolation of *F. graminearum* from soil. The efficacy of different types of selective media was also compared for the isolation of *F. graminearum* from wheat debris. 

## 2. Materials and Methods

### 2.1. Fusarium graminearum Strains

Three strains of *F. graminearum* (FG2556, FG2498, FG2481) were isolated from Fusarium head blight-infected wheat samples collected in 2016 and were supplied by Dr. Phil Jennings, Fera Science Ltd. (York, UK). All strains were confirmed as *F. graminearum* using species-specific PCR [19].

### 2.2. Czapek Dox Propiconazole Dichloran Agar

CZID media [6] was modified to Czapek Dox propiconazole dichloran agar (CZPD) as described by Hofgaard et al. [20]. The CZPD media contained (per L distilled water): 48 g of Czapek Dox agar (Sigma-Aldrich, Buchs, Switzerland), 1 mL of 0.2% dichloran (Aldrich, Steinheim, Germany) solution in ethanol, 1 mL of 5% chloramphenicol (Sigma, Shanghai, China) solution in ethanol, 1 mL of trace metal solution (1 g ZnSO_4_·7H_2_O [Fisher Scientific, Loughborough, UK] + 0.5 g CuSO_4_·5H_2_O [Fisher Scientific] per 100 mL distilled water), 10 mL of filter-sterilised 0.5% chlortetracycline hydrochloride (Sigma, Rimini, Italy) solution and 1 mL of 0.3% *Bumper*^®^ suspension (containing 750 µg propiconazole). Chlortetracycline and *Bumper*^®^ solution were added after autoclaving and cooling the media to 55 °C.

### 2.3. Preliminary Screening of Fungicides

Preliminary work involved the screening of five fungicides, namely azoxystrobin, boscalid, pyraclostrobin, fluxapyroxad and prochloraz, for their potential as selective inhibitors in culture media for the isolation of *F. graminearum* from soil. Soil suspension (0.1%, 1%, 10% *w*/*v*), *F. graminearum* mycelial plugs and conidial suspensions were separately inoculated on PDA amended with these fungicides at concentrations ranging from at 0.001 to 1000 mg·L^−1^. This was conducted to identify effective concentrations of fungicides that would allow the growth of *F. graminearum* whilst suppressing the growth of other fungi. Based on the results with amended PDA, fluxapyroxad and pyraclostrobin were selected for further screening and tested at concentrations ranging from 0.001 to 100 mg·L^−1^ in amended CZPD as described above.

### 2.4. Isolation of Fusarium graminearum from Soil Using CZPD with and without Fluxapyroxad

Based on the preliminary screening, fluxapyroxad was selected for further testing. CZPD media was prepared as described above and another set of CZPD media was amended with fluxapyroxad at a 1 mg·L^−1^ concentration after autoclaving and cooling the media to 55 °C. A stock solution of *F. graminearum* (10^5^ spores mL^−1^) was prepared as described previously [21] and diluted in a 10-fold serial dilution using sterile distilled water and soil suspension. The soil suspension was prepared by adding 5 g of fresh soil (soil collected from Harper Adams University estate, UK) to sterile distilled water to give a final volume of 50 mL and mixed well. Aliquots of the soil suspension and sterile distilled water were used to dilute the conidia stock solution to 10^4^, 10^3^ and 10^2^ spores mL^−1^. Triplicate plates of CZPD and fluxapyroxad-amended CZPD were spread with 100 µL of the dilutions. Later colony-forming units (cfu) were counted on the plates after 5–8 days of incubation at room temperature (ca. 18 °C). Growth of *F*. *graminearum* in plates spread with dilutions from the soil suspension was recorded based on the characteristic reddish pink pigmentation. A subset of ca. 10% of assumed *F. graminearum* colonies were sub-cultured on PDA media plates and incubated at room temperature (ca. 18 °C) for 14 days. The conidia were harvested as described by Ashiq et al. [21] and confirmed as *F. graminearum* based on spore morphology [22].

### 2.5. Comparing Selectivity of Media Using Fusarium graminearum-Infected Wheat Debris

Four types of media were compared for their selective efficacy to isolate *F. graminearum* from wheat debris. CZPD and CZPD amended with 1 and 5 mg·L^−1^ fluxapyroxad were prepared as described above, while Komada’s media was prepared as described in Komada [3]. Wheat debris (chaff, straw, rachis) was collected post-harvest from a *F. graminearum*-inoculated wheat field experiment at the research facilities of Harper Adams University, Newport, Shropshire, UK. The debris were surface sterilised with sodium hypochlorite (1.2% available chlorine) containing 0.05% Tween 20 for 3 min and washed three times with sterile distilled water. Five debris pieces were placed per plate in five plates of each of the four media types. Plates were incubated at room temperature (ca. 18 °C) and *F. graminearum* growth was observed after 7–14 days. *Fusarium graminearum* colonies were confirmed as described above. 

## 3. Results

### 3.1. Isolation of Fusarium graminearum from Soil Using Fluxapyroxad

**Figure 1:**
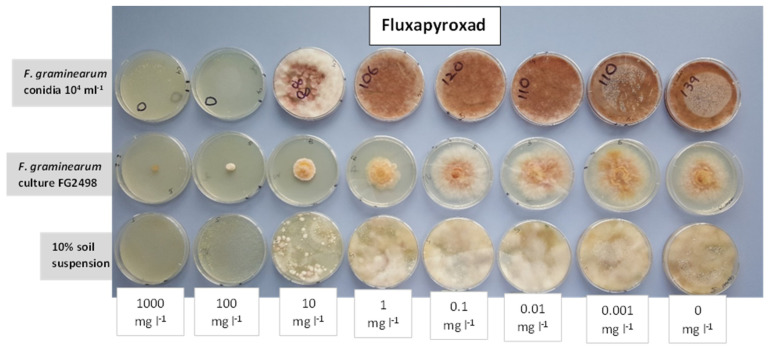
Different concentrations of fluxapyroxad added to potato dextrose agar (PDA) and tested for their potential as a selective inhibitor for the development of a selective medium for *Fusarium graminearum*. The growth of *F. graminearum* from a conidial suspension (10^4^ mL^−1^), a mycelial plug from culture FG2498 and growth from 10% soil suspension on (from right) PDA-unamended, fungicide a.i at 0.001, 0.01, 0.1, 1, 10, 100, 1000 mg·L^−1^ PDA after 5 days of incubation at room temperature (ca. 18 °C).

**Figure 2:**
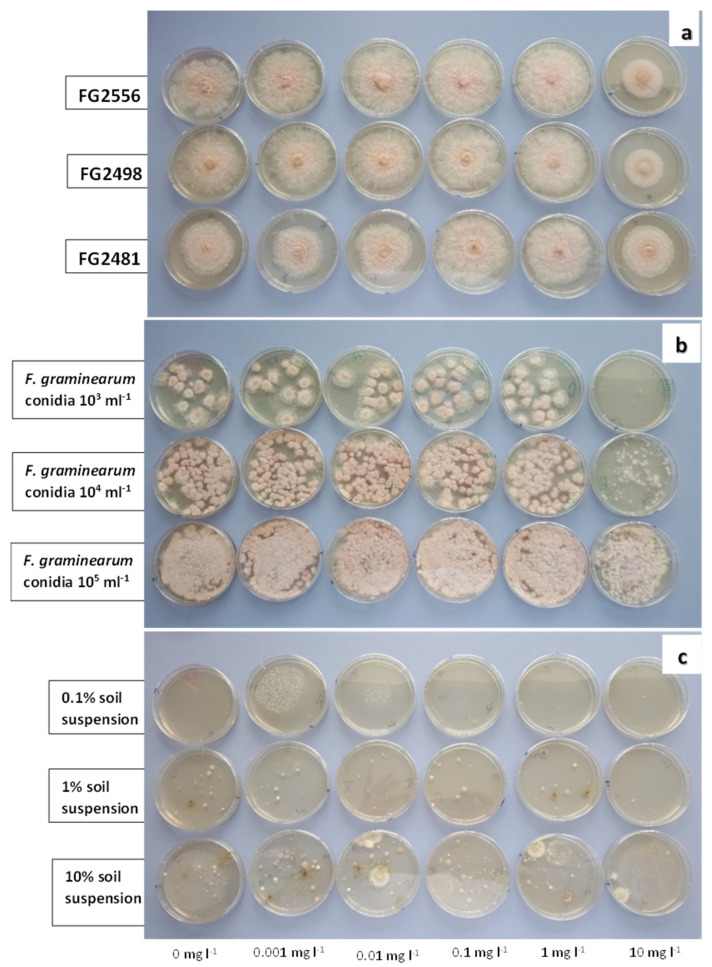
Different concentrations of fluxapyroxad added to Czapek Dox propiconazole dichloran agar (CZPD). (**a**) Growth of *Fusarium graminearum* culture from mycelial plug, (**b**) growth of *F. graminearum* from conidial suspension and (**c**) growth from soil suspension on (from left) CZPD-unamended, fungicide a.i at 0.001, 0.01, 0.1, 1, 10 mg·L^−1^ CZPD after 5 days of incubation at room temperature (ca. 18 °C).

Preliminary results from media amended with different concentrations of the five fungicides (azoxystrobin, boscalid, pyraclostrobin, fluxapyroxad, prochloraz) suggested that fluxapyroxad could be a potential inhibitor to isolate *F. graminearum* from soil (Figure 1 and Figure 2). The mean of values for colony counts of *F. graminearum* on unamended CZPD and fluxapyroxad-amended CZPD media after 8 days of incubation are presented in Table 1. Colony counts of *F. graminearum* were similar on the two types of media used. Additionally, the *F. graminearum* colony count, on both types of media, was similar when conidia were introduced with either the sterile distilled water or soil suspension. However, neither medium proved to be particularly selective as many other soil fungi were able to grow well on both media.

### 3.2. Selectivity of Media for Isolation of Fusarium graminearum from Wheat Debris

**Figure 3:**
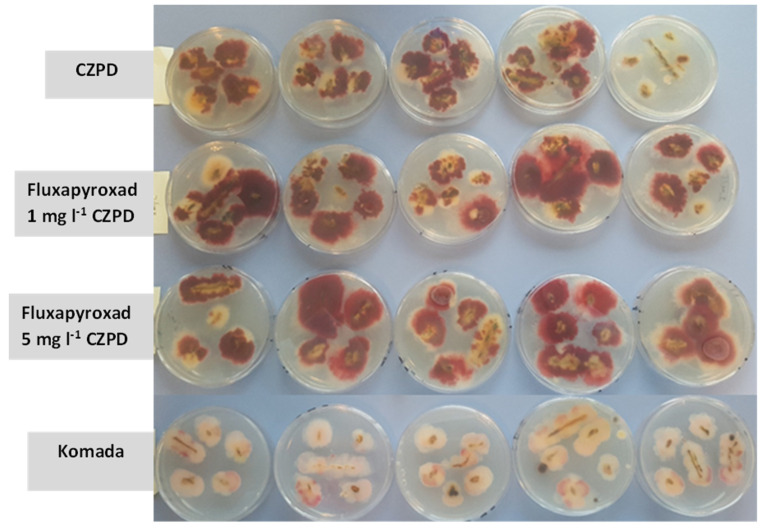
*Fusarium graminearum*-infected wheat debris on different media after 10 days (from the top: Czapek Dox propiconazole dichloran agar (CZPD); fluxapyroxad 1 mg·L^−1^ CZPD; fluxapyroxad 5 mg·L^−1^ CZPD; Komada’s media).

When wheat debris was used, there was little difference between CZPD, fluxapyroxad amended-CZPD and Komada’s media in terms of selectivity. *Fusarium graminearum* colonies became identifiable and produced a dark pigmentation on CZPD (both unamended and amended) after 9–10 days (Figure 3). The colour of *F. graminearum* colonies on Komada’s media became darker and identifiable after 17–18 days, yet not as dark as observed on CZPD after 10 days. CZPD media also appeared to have a less inhibitory effect on *F. graminearum* growth.

## 4. Discussion

The current selective media for the isolation of *Fusarium* are based on old fungicide chemistry, while *F. graminearum* lacks sensitivity towards newer fungicides such as strobilurins and SDHI. Hence, it was of interest to investigate whether these newer fungicides would improve the selectivity of *Fusarium* media. However, the fungicides tested did not prove to be effective inhibitors in culture media for the isolation of *F. graminearum* from soil. CZPD and fluxapyroxad-amended CZPD allowed the growth of *F. graminearum* conidia in the soil suspension but they did not suppress other fungi in the soil samples. 

An ideal selective medium for a specific group of fungi should promote the growth of all viable propagules of this specific fungal group, facilitate its identification and restrict the growth of unwanted microbiota. Moreover, the stability and toxicity of the added inhibitors are important factors that should be considered for the efficacy and safety of the culture medium [23]. Widely used media such as Nash and Snyder [1] and Komada’s medium [3] contain PCNB as a fungal inhibitor. Although PCNB has been categorised as "not classifiable as to its carcinogenicity to humans" by the International Agency for Research on Cancer [24], the US Environmental Protection Agency has classified it as possible human carcinogen (Group C) [25]. Malachite Green is used to replace PCNB in culture media but the carcinogenic properties of Malachite Green are evident in experimental animals [26,27]. Although, Malachite Green has recently been classified in Group 3 as “not classifiable as to its carcinogenicity to humans” by the International Agency for Research on Cancer [28], however, previous studies suggest that Malachite Green is a multi-organ toxin [29]. Another drawback of this dye is that it is deactivated upon exposure to light which might affect its antifungal activity [30]. 

CZID media was recommended by Thrane [31] to be used for the detection of *Fusarium* in food samples, as the colony morphology of *Fusarium* on this medium allowed the easier identification of different *Fusarium* species. The originally published CZID media [6] is here modified to CZPD [20] where iprodione is replaced with propiconazole to make the media more stable and long lasting. Abildgren et al. [6] used 3 mg of iprodione per L of CZID media, whereas in CZPD, 750 µg of propiconazole per L of media is used instead.

In an attempt to improve selectivity, a higher concentration of fluxapyroxad (5 mg·L^−1^) was also tested when comparing media using the *F. graminearum*-infected wheat debris. However, no observable differences were seen between the two concentrations of fluxapyroxad (1 mg·L^−1^ or 5 mg·L^−1^) and unamended CZPD media. The widely recommended media, Komada’s, was also included for this part of the study. CZPD (both unamended and amended) was better in terms of pigmentation and growth rate, requiring a shorter incubation time of 9-10 days compared to Komada’s media. Moreover, the colonies on CZPD were easily identifiable due to their darker pigmentation. Due to the greater growth rate of *F. graminearum* on CZPD, the colonies were larger compared to those on Komada’s media, suggesting CZPD has a less inhibitory effect on *F. graminearum* growth. CZPD medium has been successfully used in our laboratory to isolate *F. graminearum* from plant material, providing a more reliable assessment than the widely used Komada’s media. However, these media are not sufficiently selective when isolating *F. graminearum* from complex fungal populations such as soil where many other fungi are able to grow on these media. Therefore, further studies are required to develop a more selective medium for the isolation of *F. graminearum* from soil.

## Figures and Tables

**Table 1:** Colony-forming units of *Fusarium graminearum* from three dilutions of *F. graminearum* conidia (10^4^, 10^3^, 10^2^ spores mL^−1^) in sterile distilled water and soil suspension on Czapek Dox propiconazole dichloran agar with and without fluxapyroxad. Data given are the mean from triplicate plate counts and numbers in parentheses represent the standard error of the mean.

Media	Diluent	Colony-Forming Units Plate^−1^					
		10^4^		10^3^		10^2^	
		Fg ^a^	Other ^b^	Fg	Other	Fg	Other
CZPD ^c^	Sterile distilled water	24 (3) ^d^	0	2 (1)	0	1 (1)	0
	Soil suspension	28 (2)	48 (2)	7 (2)	58 (7)	3 (1)	55 (5)
fluxapyroxad-CZPD ^d^	Sterile distilled water	32 (4)	0	3 (1)	0	1 (1)	0
	Soil suspension	26 (2)	49 (5)	10 (2)	61 (7)	2 (1)	70 (3)

^a^*Fusarium graminearum*. ^b^ Other fungal species. ^c^ Czapek Dox propiconazole dichloran agar. ^d^ CZPD amended with 1 mg·L^−1^ fluxapyroxad.

## Data Availability

All data generated or analysed during this study are included in the published article.
